# Expression of *CdDHN4*, a Novel YSK_2_-Type Dehydrin Gene from Bermudagrass, Responses to Drought Stress through the ABA-Dependent Signal Pathway

**DOI:** 10.3389/fpls.2017.00748

**Published:** 2017-05-16

**Authors:** Aimin Lv, Nana Fan, Jianping Xie, Shili Yuan, Yuan An, Peng Zhou

**Affiliations:** ^1^School of Agriculture and Biology, Shanghai Jiao Tong UniversityShanghai, China; ^2^Key Laboratory of Urban Agriculture (South), Ministry of AgricultureShanghai, China

**Keywords:** *Cynodon dactylon*. L, dehydrin, drought, promoter, ABA

## Abstract

Dehydrin improves plant resistance to many abiotic stresses. In this study, the expression profiles of a dehydrin gene, *CdDHN4*, were estimated under various stresses and abscisic acid (ABA) treatments in two bermudagrasses (*Cynodon dactylon* L.): Tifway (drought-tolerant) and C299 (drought-sensitive). The expression of *CdDHN4* was up-regulated by high temperatures, low temperatures, drought, salt and ABA. The sensitivity of *CdDHN4* to ABA and the expression of *CdDHN4* under drought conditions were higher in Tifway than in C299. A 1239-bp fragment, CdDHN4-P, the partial upstream sequence of the *CdDHN4* gene, was cloned by genomic walking from Tifway. Bioinformatic analysis showed that the CdDHN4-P sequence possessed features typical of a plant promoter and contained many typical *cis* elements, including a transcription initiation site, a TATA-box, an ABRE, an MBS, a MYC, an LTRE, a TATC-box and a GT1-motif. Transient expression in tobacco leaves demonstrated that the promoter CdDHN4-P can be activated by ABA, drought and cold. These results indicate that *CdDHN4* is regulated by an ABA-dependent signal pathway and that the high sensitivity of *CdDHN4* to ABA might be an important mechanism enhancing the drought tolerance of bermudagrass.

## Introduction

Dehydrins (group-II late embryogenesis abundant proteins) accumulate in embryogenesis during seed maturation and in vegetative tissue in response to drought, salinity or cold (Dure et al., 1981; Close, 1996; Hara et al., 2013). Generally, these proteins are hydrophilic proteins that contain three conserved motifs: the K, Y, and S segments (Rorat, 2006; Eriksson et al., 2011; Hara et al., 2011). The K segment is similar in sequence to EKKGIMDKIKEKLPG, which is a common feature of all dehydrins (DHNs) and a Lys- rich motif that forms an α-helix in the C-terminus that may participate in interaction with membranes and proteins (Allagulova et al., 2003; Koag et al., 2009; Hughes et al., 2013). DHNs have been subdivided into five subclasses according to the conservative segments: Y_n_SK_n_, K_n_, SK_n_, Y_n_K_n_, and K_n_S (Close, 1997; Hundertmark and Hincha, 2008; Hara et al., 2009; Brini et al., 2011; Eriksson et al., 2011). These DHNs respond to different environmental factors (Allagulova et al., 2003; Rorat, 2006). Many studies have shown that expression of Y_n_SK_n_ dehydrins is mainly induced by dehydration, while K_n_, K_n_S, SK_n_, and Y_n_K_n_ dehydrins are up-regulated by cold (Graether and Boddington, 2014). Phytohormones are vital factors that affect dehydrin expression. For example, Y_n_SK_n_ and Y_n_K_n_ dehydrins are induced by ABA (Battaglia et al., 2008). However, some DHNs do not accumulate in plants treated with ABA (Whitsitt et al., 1997). Grossi found that the barley dehydrin genes *paf93* (a cold-regulated gene) and *cdr29* (a cold-desiccation responsive gene) were regulated by low temperatures and drought stress, but not by exogenous ABA (Grossi, 1995). Therefore, the expression of dehydrin genes occurs via ABA-dependent and ABA-independent pathways (Allagulova et al., 2003).

ABA is a phytohormone that performs several specific functions in plant growth and development. Drought, cold and salt stresses can cause an increase in biosynthesis and accumulation of ABA, which can be rapidly catabolized following the relief of stress (Taylor et al., 2000). Increases of ABA levels are always accompanied by major changes in gene expression and adaptive physiological responses (Zeller et al., 2009). Among ABA-induced genes, dehydrins are important in plant resistance to stress (Graether and Boddington, 2014; Tuteja, 2014). Many DHNs have been identified in plants including *Arabidopsis thaliana, Citrus unshiu*, wheat, and *Vigna radiata* (Close, 1997; Koag, 2003; Hara et al., 2009; Lin et al., 2012). Most are up-regulated by exogenous ABA. Although functions of DHNs remain unclear, the accumulation of DHNs during cell dehydration is involved in a protective response.

The regulation of genes under various stress conditions is associated with interactions between *cis*-acting elements and transcription factors (TFs) (Bassett et al., 2009). Many *cis*-acting elements of DHN promoters in plants respond to one or several environmental signals (Lee et al., 2013; Zhu et al., 2014). These *cis*-acting elements include ABA-responsive elements (ABREs), drought-responsive elements/C-repeats (DRE/CRT), low-temperature-responsive elements (LTRE) and light-responsive elements. For example, Zhu et al. found that the *wzy1-2* promoter, a promoter of the SK_3_-type dehydrin gene, contained several elements, including ABRE, LTRE, the gibberellin (GA)-responsive element (in wheat) and that *wzy1-2* could be induced by cold or ABA, suggesting that different *cis*-acting elements are vital in gene expression under stress (Zhu et al., 2014).

The accumulation of dehydrin in bermudagrass (*Cynodon dactylon* L.) plays an important role in adaption to environmental stress. Many dehydrin proteins and genes associated with drought tolerance in bermudagrass had been reported. Kemin Su confirmed that the expression of 16- and 23-kDa dehydrin are associated with drought tolerance in bermudagrass (Su et al., 2013). Hu et al. suggested that the accumulation of 31- and 40-kDa dehydrins in bermudagrasses might contribute to their drought tolerance (Hu et al., 2010). Zhou identified a dehydrin gene in bermudagrass Tifway with high sequence identity to the DHN4 gene of barley (*Hordeum vulgare*). The dehydrin gene was up-regulated when Tifway was exposed to drought for 10 days, and its expression level was significantly higher in Tifway (drought-tolerant) than in C299 (drought-sensitive) (Zhou et al., 2014). Thus, studying the expression and regulation of dehydrin genes under different abiotic stress conditions contributes to our understanding of the resistance of bermudagrass to abiotic stresses. We determined the full-length cDNA sequences of a novel YSK_2_-type dehydrin *CdDHN4* from Tifway (drought-tolerant) and C299 (drought-sensitive) (GenBank accession no. KX243552).

The objective of this study was to determine the regulation of dehydrin genes in drought conditions. The main steps were as follows: (1) isolating the promoter sequence of *CdDHN4* and analyzing its *cis*-acting elements in bermudagrass; (2) identifying environmental stresses that can activate the *CdDHN4* promoter and inducing the *CdDHN4* expression; (3) the response of *CdDHN4* expression under drought and ABA conditions in Tifway and C299.

## Materials and methods

### Plant materials and treatments

The hybrid bermudagrass (Tifway) and common bermudagrass (C299) used in this study, were collected from 3-year-old sod from the turfgrass at Shanghai Jiao Tong University, Shanghai, China. The plants were grown in a plastic pot (20 cm in diameter and 40 cm in height) filled with sand. The plants were maintained in a growth chamber with a temperature regime of 30/25°C (day/night), a 14-h photoperiod, 70 ± 5% relative humidity and a photosynthetically active radiation of 480 mmol m^−2^ s^−1^ at the canopy level. The plants were irrigated three times per week until field capacity was reached and fertilized fortnightly with 1/2 Hoagland's. Turfgrass was maintained under the above conditions for 40 d to establish a turf canopy and root systems with a plant height of about 6 cm.

The stress experiments consisted of four treatments: well-watered control, drought stress, cold treatment, heat treatment and salt treatment. Each treatment included three biological replicates. All containers were relocated every other day inside the chamber. The well-watered control plants were maintained in the chamber as described above. Drought stress was induced by withholding irrigation for 15 days. Cold stress was induced by transferring plants to a temperature of 10°C/5°C (day/night) for 15 days. Heat stress was induced by incubating plants in a growth chamber at 45°C/40°C (day/night) for 15 days and watered twice a day. Salt stress was induced by watering the plants every 2 days with 200 mM NaCl for 5 days. The relative water content (RWC), photochemistry efficiency (Fv/Fm) and cell-membrane stability in the leaves were measured in each treatment. The leaves were sampled at 0, 3, 6, 9, 12, and 15 days after treatment. Approximately 100 mg leaves were sampled and rapidly frozen in liquid nitrogen, and stored at −80°C until use. RWC was measured using 10–15 fully expanded leaves according to the Weatherley methods (Barrs and Weatherley, 1962; Hu et al., 2010). Photochemistry efficiency was measured with a chlorophyll fluorescence spectrometer (OS1-FL, USA). Cell-membrane stability was determined by electrolyte leakage (EL). Electrolyte leakage (EL) was measured with a conductance meter by DaCosta's method (DaCosta et al., 2004; FE30, METTLER TOLEDO, Switzerland).

For tissue-level expression analysis, Tifway and C299 were grown without water for 15 days. The plant leaves, stems and roots were, respectively, sampled at 0, 5, 10, and 15 d during the drought treatment. Each treatment included three independent replicates.

Exogenous ABA treatment was conducted under normal growth conditions. The plants were sprayed with 0, 5, or 50 μM ABA solution containing 0.05% Tween20 (v/v) and sampled at 0, 6, 12, and 24 h after treatment.

### RNA extraction and transcript-accumulation analysis of *CdDHN4* with real-time PCR

Total RNA was prepared from 100 mg plant material (leaves, stems and roots) using Trizol Reagent (Life Technologies, USA). Contaminating genomic DNA was removed by incubating the total RNA with RNase-free DNase (Promega) at 37°C for 30 min. First-strand cDNA synthesis was carried out using 1 μg total RNA and the PrimeScript™ 1st Strand cDNA Synthesis Kit (TaKaRa, Dalian, China) according to the manufacturer's recommendation.

Eash real-time PCR reaction (20 μL) contained 1 μL cDNA. Amplification was performed with SYBR® Premix ExTaq™ II (TaKaRa), following the manufacturer's protocol. The primer sequences (*CdDHN4*-F/*CdDHN4*-R, 18S as an internal control) are provided in Table 1. The cycling conditions were 5 s at 95°C, 40 cycles of amplification at 95°C for 5 s, at 59°C for 10 s and extension at 72°C for 30 s. The PCR reactions were run in an Agilent Mx3000 Real Time PCR Detection System. The relative expression was determined using the 2^−ΔΔCT^ method (Pfaffl, 2001). Three biological replicates were examined.

**Table 1:** **Primer sequences used in experiments**.

**Primer ID**	**Fordward primers (5′−3′)**	**Reverse primers (5′−3′)**
D1	ATGGAGCACCAGGGACAGTACGGCC	CATGCCCATTCCTCCTGTTC
D2	GAACAGGAGGAATGGGCATG	GGAGCTTTTCCTTGATCTTATCCT
D3	CCGGAGGAAGAAGGGAATCA	GGAGCTTCTCCTTGATCTTGTC
D4	GGCATAATGGACAAGATCAAGGA	GACAAAAGACACTTAATATATGTATTTCAGA
D5	ATGGAGCACCAGGGACAGTACGGCC	GGAGCTTCTCCTTGATCTTGTC
PR3		GTTCCCATGCCCATTCCTCCTGTTCC
PR2		CGGTGGTGCCGTGGGTTCCC
PR1		CCCATGCCGCCGGTACCGGT
AP1	GTAATACGACTCACTATAGGGC	ACCATACAGACCCTTCAGAC
CdDHN4	GCGAACAGTCCGTGATAACT	GACACTAATGCGCCCGGTAT
18S	GTGACGGGTGACGGAGAATT	
TIF-P	GTAGTTTCGTCTGAGTGTTGCG	GGTGACTACTGACTACTAAGCTG
TIF-EP	CAAGCTTGTAGTTTCGTCTGAGTGTTGCG	GGAATTCGGTGACTACTGACTACTAAGCTG

*The underlined represents restriction enzyme sequence*.

### Cloning genomic DNA and the promoter of *CdDHN4* from Tifway and C299

Genomic DNA was extracted from leaves as described in the TIANGAN User Manual. The genomic sequence of *CdD*HN4 gene was amplificated with primers (D1F/D1R, D2F/D2R, D3F/D3R, and D4R/D4F) using *TaKaRa Ex Taq*® (Table 1).

The 5′- and 3′-flanking regions of *CdDHN4* were cloned by genomic walking using the Universal Genome Walker 2.0 kit (Cloneth, Italy). Genomic DNA was digested separately with four blunt-end restriction enzymes (*Dra*I, *EcoR*V, *Pvu*II, and *Stu*I), and the products were purified using NucleoSpin Gel and PCR Clean-Up kit. The digested, purified DNA was incubated at 16°C overnight with Genome Walker Adaptor (25 μM) and at 70°C for 5 min to stop the reactions to produce four libraries.

Based on the genomic sequence already obtained, gene-specific primers (PR1, PR2, and PR3; Table 1) and AP1 primer (outer Adapter Primer, provided by the kit) were used to amplify the 3′- and 5′-flanking regions, respectively, with the Advantage 2 Polymerase Mix (Clontech). Hot-start and touch-down PCR were used with primary and secondary PCR (nested PCR). Thermal cycling for primary PCR was as follows: 7 cycles at 94°C for 25 s and 72°C for 3 min, follows by 32 cycles at 94°C for 25 s and 67°C for 3 min and 67°C for an additional 7 min after the final cycle. For the secondary PCR, using the diluted primary PCR product as templates, thermocycling was performed as 5 cycles of 94°C for 25 s and 72°C for 3 min, followed by 20 cycles of 94°C for 25 s and 67°C for 3 min, with a final extension at 67°C for 7 min. PCR products were sequenced by Sangon Biotech (Shanghai, China). All these DNA fragments were assembled to complete the promoter sequence of *CdDHN4*. Putative functional elements of the promoter were identified by the PLACE database (Higo et al., 1999) and PlantCARE database (Lescot et al., 2002).

Different primer pairs of *CdDHN4* were used for gene amplification to ensure that the assembled gene was not a hybrid gene containing segments from other dehydrin genes (Zhou et al., 2011). After sequencing, the resulting DNA fragments were aligned with the assembled sequence. All sequence information was integrated, and we confirmed that we had obtained the whole unique sequence of the *CdDHN4* gene, including its promoter sequence. The whole 5′-flanking region of *CdDHN4* approximately 1239 bp, was amplified using the primer pair TIF-P(F/R) and linked into the pMD18-T plasmid Prodhn4-pMD18-T, then sequenced.

### Vector construction and transient assays in *Nicotiana benthamiana*

The vector pBI121 GUS reporter gene was substituted for the green fluorescent protein (GFP) gene with the restriction enzymes *BamH*I/*EcoR*I to generate PB35S::GFP. An upstream region of *CdDHN4*, named Prodhn4, was amplified using the Prodhn4-pMD18-T plasmid as a template and with the primers TIF-EP (F/R), and added *Hind*III/*EcoR*I restriction sites. The amplification product was digested with *Hind*III/*EcoR*I, cloned into PB35S::GFP reporter vector that was digested with the *Hind*III/*EcoR*I to remove the CaMV35S promoter. The recombined vector PBCdDHN4-P::GFP was sequenced. For transient plant transformation, the binary vector were transformed into *Agrobacterium tumefaciens* (GV3101) and infiltrated with a 1 ml syringe without needle into fully expanded leaves of tobacco (*N. benthamiana*), as described by Witte et al. (2004). As a negative control, tobacco leaves were infiltrated with the empty binary vector pBI121. After 48 h in growth chamber with 25°C, 75% relative humidity, darkness. The leaves were washed twice and observed in low light using a Leica spectral confocal microscope (Leica SP5, Leica Microsystems, Wetzlar, Germany), with an excitation wavelength of 488 nm. To determine whether the promoter was ABA, cold or drought inducible, the tobacco were suffered from 50 μM ABA, 4% PEG or transferred 4°C for 12 h before scanning.

### Quantitation of ABA in bermudagrass leaves

An indirect competitive enzyme-linked immunosorbent assay (icELISA) was applied to quantitate the changes in levels of ABA in the leaves of the two bermudagrasses under ABA or drought treatments (You-Ming et al., 2001).

### Statistical analysis

ANOVA was performed with SAS10.0 software. Data were expressed as the mean ± SD. Pairwise differences among treatments were tested using a mean separation test named least significant difference (LSD) at the *P* = 0.05 level.

## Results

### Physiological response of bermudagrass to abiotic stresses

There were no significant differences in soil water content (SWC) between Tifway and C299 on days 5, 10, and 15 of drought stress (*p* < 0.05), which confirmed that the drougt conditions for the two bermudagrasses were the same. Relative water content (RWC) declined more dramatically in C299 leaves than in Tifway leaves, and on days 5, 10, and 15 of drought stress, RWCs were significantly lower in C299 than in Tifway (*p* < 0.05). Additionally, electrolyte leakage (EL) was significantly higher in C299 leaves than in Tifway leaves at days 5, 10, and 15 (Table 2).

**Table 2:** **Variation in soil water content (SWC), leaf relative water content (RWC), and electrolyte leakages (EL) for bermudagrass under drought stress**.

**Index**	**Varieties**	**Control**	**Drought for 5 d**	**Drought for 10 d**	**Drought for 15 d**
SWC (%)	Tifway	18.0 ± 4.8A	10.0 ± 2.2A	2.5 ± 0.4A	0.5 ± 0.1A
	C299	16.9 ± 1.7A	8.3 ± 2.4A	2.1 ± 0.2A	0.3 ± 0.02A
RWC (%)	Tifway	91.9 ± 2.2a	90.6 ± 1.1a	83.1 ± 2.6a	42.1 ± 1.1a
	C299	92.6 ± 2.1a	85.9 ± 1.8b	41.3 ± 3.2b	15.8 ± 0.7b
EL (%)	Tifway	9.0 ± 1.6A	11.6 ± 1.9B	28.4 ± 1.3B	70.5 ± 1.9B
	C299	8.4 ± 1.7A	21.0 ± 2.5A	63.6 ± 2.3A	86.6 ± 6.0A

*Different capital letters or small letters represent significant difference in the same columns (p < 0.05)*.

**Figure 1:**
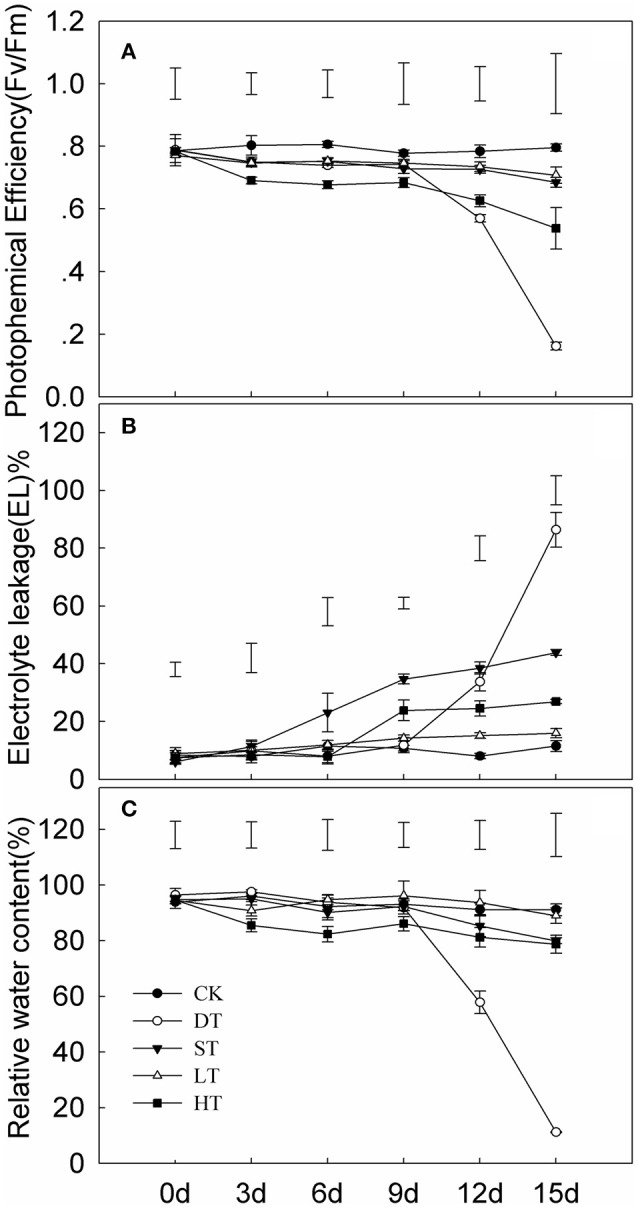
**Variation in leaf photochemical efficiency (A)** electrolyte leakage **(B)** and relative water content **(C)** of Tifway under different treatments. As a control (CK), plants were maintained under normal conditions. The drought treatment (DT) was performed by growing plants in sand without water for 15 days. The cold treatment (LT) was performed by transferring plants to a temperature of 10°C/5°C (day/night). For the heat treatment (HT), plants were incubated in a growth chamber at 45°C/40°C (day/night) and watered twice a day. For the salt treatment (ST), the plants were watered every 2 days with 200 mM NaCl for 5 days. The plant leaves were harvested at 0, 3, 6, 9, 12, and 15 d after treatments. Each treatment included three biological replicates. Vertical bars on the top indicate LSD-values (*p* < 0.05) for the comparison between treatments at a given day of treatment.

Tifway responded to drought, salt, cold and heat (Figure 1). Significant differences in photochemical efficiency (Fv/Fm), EL and RWC under four abiotic stresses were seen after day 9 of treatment. The Fv/Fm and RWC were dramatically decreased after 10 d of drought in comparison with the control, while EL was significantly increased. The greatest differences in Fv/Fm, EL, and RWC occurred under drought stress compared with the other three treatments.

### Expression of *CdDHN4* in response to abiotic stress and ABA addition

**Figure 2:**
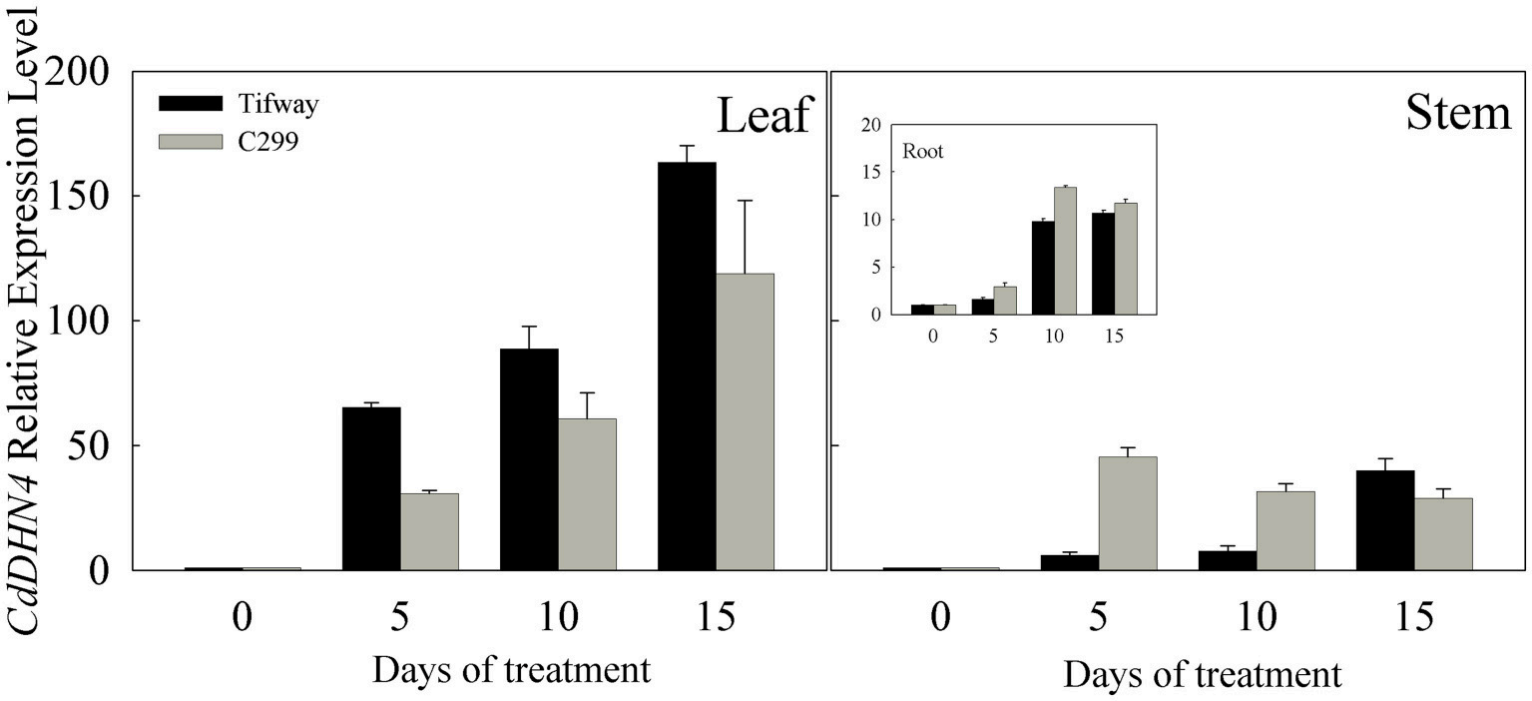
**Expression patterns of *CdDHN4* in response to drought treatment**. RT-PCR expression analysis of the dehydrin gene *CdDHN4* in leaves, syems or roots of Tifway (black columns) and C299 (dark gray columns) under water stress. Samples were collected after 5, 10, and 15 d of drought, respectively. Relative expression after normalization with 18S. The data are means of three replicates (±SE).

*CdDHN4* was up-regulated by drought in the leaves, stems and roots of both Tifway and C299, especially in leaves (Figure 2), while *CdDHN4* expression in leaves was significantly higher in Tifway than C299 at 5, 10, and 15 d, respectively.

**Figure 3:**
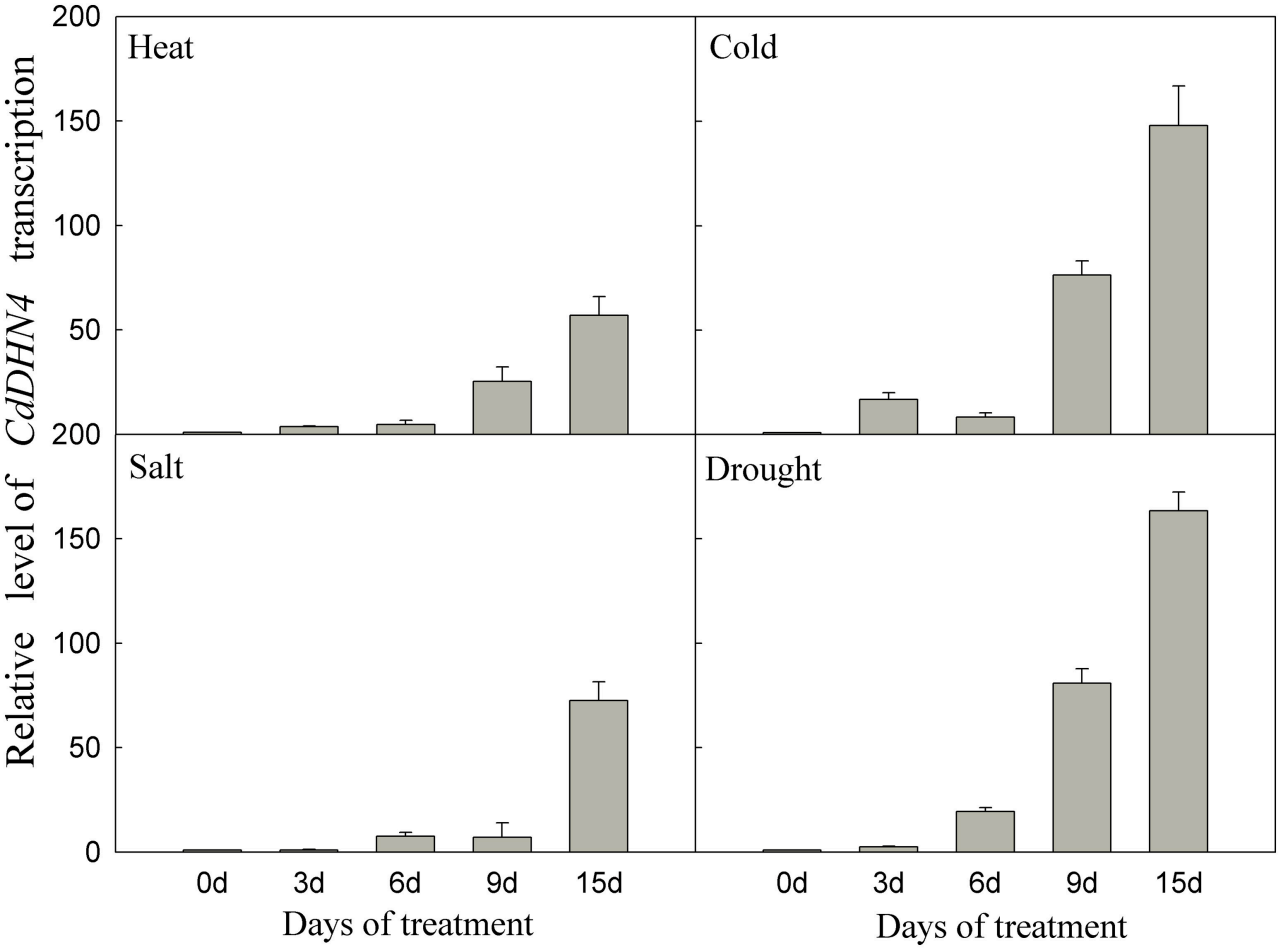
**The relative expression level of *CdDHN4* in Tifway leaves during heat, cold, salt, and drought treatments**. Five treatments were carried out as described in Figure 2. Error bars represent the standard error of three replicates.

*CdDHN4* exhibited different responses to abiotic stress. *CdDHN4* in Tifway was greatly up-regulated by drought, cold, heat and salt treatments, and the *CdDHN4* expression was significantly higher under drought and cold treatments than under salt and heat treatments at 9 and 15 d (Figure 3).

**Figure 4:**
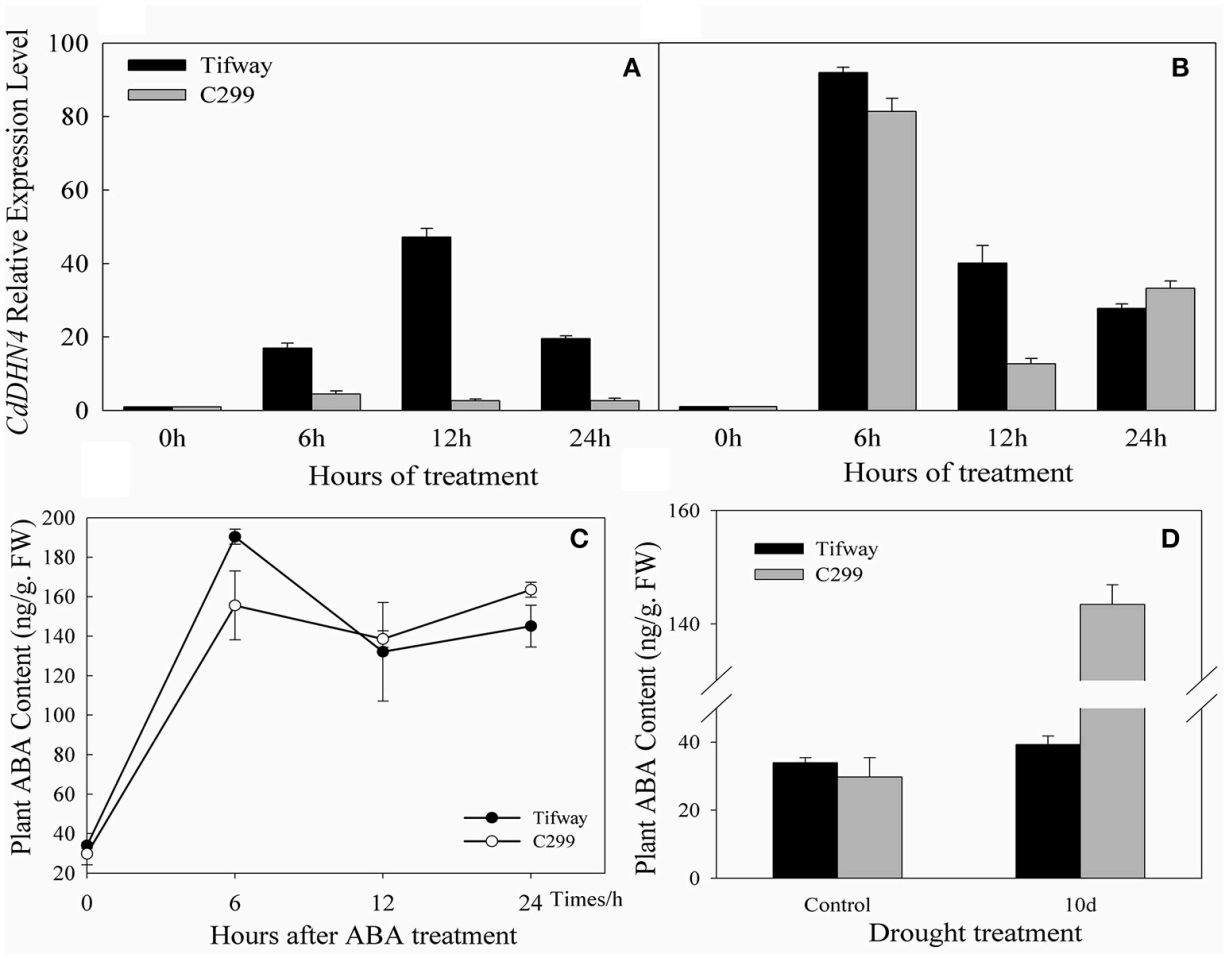
**Accumulation of *CdDHN4* transcripts and the levels of endogenous ABA in Tifway and C299 leaves in response to ABA or drought stress**. Tifway and C299 were treated with 5 μM or 50 μM ABA containing 0.05% Tween20 (v/v) and then sampled at 0, 6, 12, and 24 h after treatment. The accumulation of *CdDHN4* transcripts in Tifway (black columns) and C299 (dark gray columns) after exposure 5 μM **(A)** or 50 μM **(B)** ABA. **(C)** The level of endogenous ABA in Tifway and C299 leaves after they were sprayed with 50 μM ABA. **(D)** The level of endogenous ABA in Tifway and C299 leaves under drought stress.

Tifway and C299 were sprayed with exogenous ABA, and detached leaves were used to analyze gene expression at 0, 6, 12, and 24 h. The transcription of *CdDHN4* increased in the presence of ABA (Figures 4A,B). Under the 5 μM ABA treatment, the number of *CdDHN4* transcripts reached maximum levels at 6 h in C299 (4.52-fold higher than in the control) and at 12 h in Tifway (16.96-fold higher than in the control). The number of *CdDHN4* transcripts was significantly greater in Tifway than in C299 at 6, 12 and 24 h. Under the 50 μM ABA treatment, the relative expression levels of *CdDHN4* were only 1.23 and 3.33-fold higher in Tifway than in C299 at 6 and 12 h, respectively, but the expression level was lower in Tifway than in C299 at 24 h.

### Response of endogenous ABA to drought and ABA addition

After 50 μM ABA was sprayed on the leaves, the ABA levels in the leaves of Tifway and C299 were 190.34 ng·g^−1^ FW and 155.57 ng·g^−1^ FW at 6 h, 5.6 times and 5.22 times higher than they were at 0 h, respectively (Figure 4C). The levels of ABA in both bermudagrass genotypes decreased after 6 h, and were higher in Tifway than in C299 at 12 and 24 h.

The ABA content of leaves increased significantly after 10 d of drought treatment in comparison with the control. The level of endogenous ABA was 3.65-fold higher in C299 than in Tifway under drought stress (Figure 4D).

### Sequence analysis of the *CdDHN4* promoter

The *CdDHN4* genomic sequence, a 658-bp fragment, was obtained by genomic walking. Alignment between DNA and cDNA sequences revealed a 114-bp intron in the region +255 to +369 and two exons located in the position +1 (“A” of translation start site “ATG” as +1) to +254 (exon1) and +370 to +657 (exon2). The 5′ and 3′ untranslated regions contained 1239 and 252 bp, respectively (Supplement Figure 1B).

**Figure 5:**
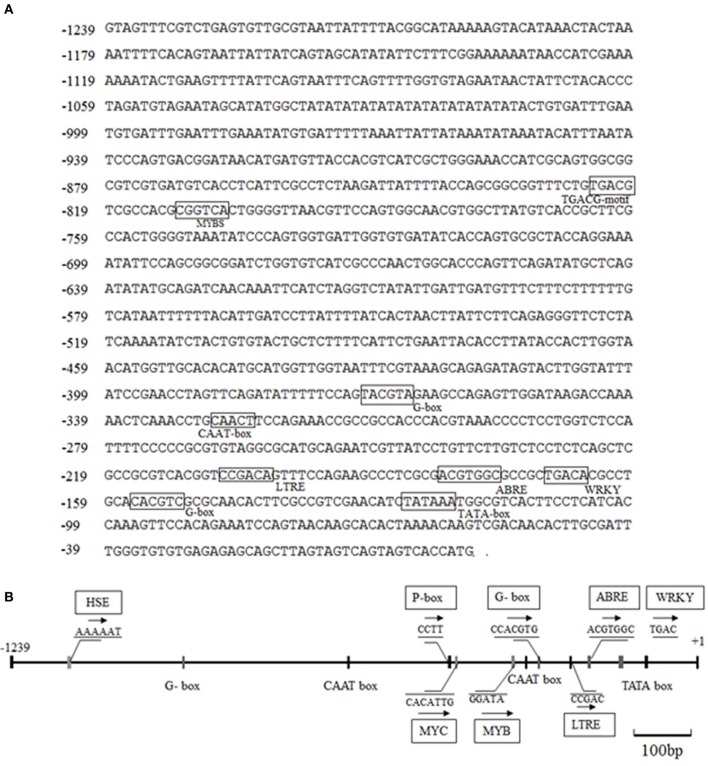
**Bioinformatic analysis of DNA sequences of the *CdDHN4* promoter**. The initiation codon ATG is indicated with +1, and putative *cis*-acting elements are shown inside boxes. The numbers on the left are the genomic DNA positions of each coding sequence. **(A)** The 5′-upstream sequence of CdDHN4. **(B)** The partially putative cis-elements in CdDHN4 promoter.

The *CdDHN4-*regulatory sequences were identified, and the 5′ upstream dehydrin was obtained. A 1239-bp genomic DNA fragment upstream of the *CdDHN4* gene, was isolated from Tifway by thermal asymmetric nested PCR. During tertiary cycling, a major specific band were amplified by PR3/AP1 primers, but not was detected by the water/AP1 primers (Supplement Figure 1A). The fragment generated with the PR3/AP1 primers (named Prodhn4) was sequenced. The sequence was identified using the PLACE and PlantCARE databases. The sequence-analysis results were consistent with features of eukaryotic promoters, including putative elements, such as a TATA box at position −35 (relative to TSS) and a CAAT box. Many *cis*-acting elements were also found in the promoter region, including ABRE, a G-box, LTRE, a TATC-box, and a CATATG- motif (Figure 5, Table 3). Several potential binding sites for transcription factors, such as MYC, MYB, WRKY and DOF-motif were also identified (Figure 5). In addition, the 5′ upstream region of *CdDHN4* was obtained from C299 using the sequence of Prodhn4 in Tifway. Sequence alignment indicated that there were only 8 bp differences between Tifway and C299 in the *CdDHN4* promoter (Supplement Figure 2). Bioinformatic analysis showed that the typical *cis* elements in C299 were consistent with those in Tifway.

**Table 3:** **Putative cis-elements in *CdDHN4* gene promoter**.

**Name**	**Consensus sequence**	**Functions**
ABRE	ACGTGGC	*cis*-acting element involved in abscisic acid responsiveness
ACE	CTAACGTATT	*cis*-acting regulatory element involved in light responsiveness
CAAT-box	CAAT	Core promoter and involved in initial transcription
CGTCA-motif	CGTCA	*cis*-acting regulatory element involved in MeJA-responsiveness
MYC	CACATG	MYC binding site
G-box	CACGTA/C	*cis*-acting regulatory element involved in light responsiveness
GC-motif	CCCCCG	enhancer-like element involved in anoxic specific inducibility
TATA-box	TATA	core promoter element around-30 from transcription start
GT1-motif	GGTTAA	*cis*-acting regulatory element involved in light responsiveness
MBS	CGGTCA/CAACTG	MYB binding site involved in drought inducibility
TA-rich region	TATATATATA	enhancer
TATC-box	TATCCCA	*cis*-acting regulatory element involved in gibberellin responsiveness
AAAG-motif	AAAG	DOF binding site
LTRE	CCGAC	*cis*-acting element involved in cold stress responsiveness
WRKY71S	TGAC	WRKY71 binding site

### Analysis of the *CdDHN4* promoter in tobacco

**Figure 6:**
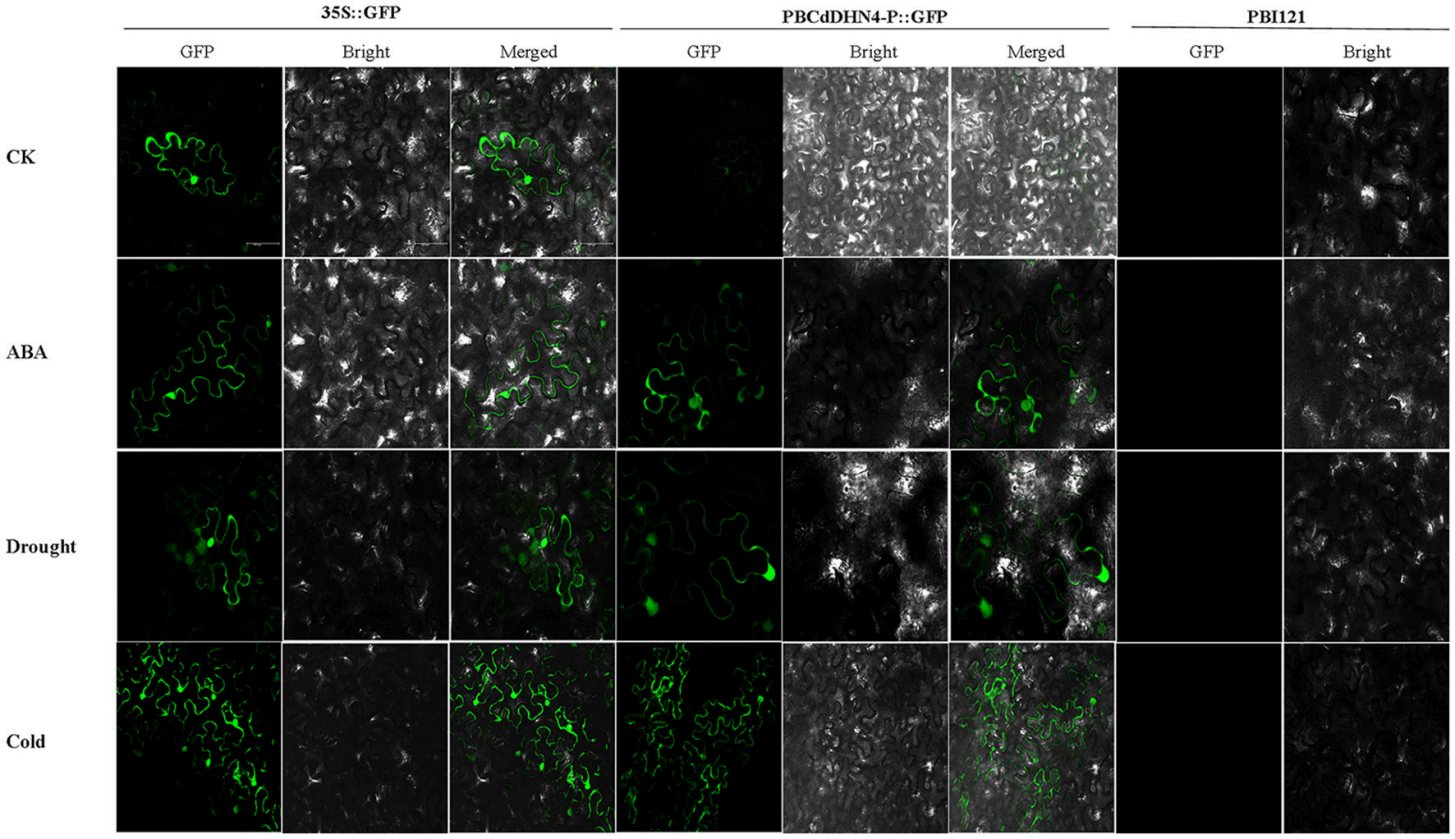
**GFP expression in tobacco leaves transformed with a dehydrin promoter under different stress treatments**. pBI121(vector only) was used as a negative control; 35S::GFP was used as a positive control; PBCdDHN4-P::GFP contains the *CdDHN4* promoter fragments. In the columns, CK represents tobacco under normal conditions; ABA, drought and cold represent treatments of 50 μM ABA, 4% polyethylene glycol 6000 and low temperatures (4°C) 12 h before observation, respectively. The scale bar corresponds to 100 μm.

The expression of GFP in tobacco leaves was observed under a Leica spectral confocal microscope. Initially, compared with empty-vector-transgenic leaves, GFP expression in PB35S::GFP and PBCdDHN4-P::GFP-transgenic leaves were similar. A similar GFP fluorescence signal was observed in tobacco leaves of PBCdDHN4-P::GFP in ABA and drought treatments, while a very weak GFP signal was observed in the control (Figure 6). Low temperatures also induced the expression of GFP.

## Discussion

The cis-active elements in promoters are crucial in the regulation of gene transcription (Biłas et al., 2016). Many dehydrin genes and their promoters have been identified, and their regulatory pathways have been well discussed (Shekhawat et al., 2011; Xing et al., 2011; Lee et al., 2013; Zhu et al., 2014; Qin and Qin, 2016). To understand the regulatory mechanisms of *CdDHN4* under different stresses, the full-length genomic and promoter sequences of a YSK_2_-type DHN were isolated and characterized from the Tifway bermudagrass cultivar. Analyzing of the 5′-flanking region of *CdDHN4* revealed that the *CdDHN4* promoter contained many putative features elements seen in other dehydrin promoters (Lee et al., 2013; Zhu et al., 2014; Qin and Qin, 2016) but also had unique features. The promoter included potential stress-responsive *cis* elements: one ABREs, two TGACG motifs, one LTRE, one MBS and one MYC. as well as different types of light-related elements (G-box, ACE, GATA motif, and Sp1). Yamaguchi-Shinozaki and Shinozaki reported that *cis*-acting regulatory elements are important molecular switches involved in the transcriptional regulation of genes responding to abiotic stress and hormone (Yamaguchi-Shinozaki and Shinozaki, 2005). ABRE is a major *cis*-acting regulatory element in ABA-dependent gene expression, and its appearance in gene promoters indicates that it is sensitive to ABA (Allagulova et al., 2003). The ABRE in the *CdDHN4* promoter contained a conserved fragment, an ACGT sequence. The promoter of the ABA-inducible gene *dhn1* in barely contains a TACGTCC fragment consisting of a G-box and GC-motif, and its removal was accompanied by loss of promoter sensitivity to ABA (Robertson et al., 1995). Amar found the *cis*-regulatory elements ABRE in wheat PrDHN-5; these elements are required for abiotic stress and ABA responsiveness (Amar et al., 2013). Rock reported that promoter sensitivity to ABA depended on a MYB element containing the TAACTG-motif and a MYC element containing the CACCTG sequence (Rock, 2000). In this study, one ABRE, one MBS and one MYC element were found near the TATA-box of the *CdDHN4* promoter, and were necessary for ABA-dependent gene expression. As a result, the *CdDHN4* promoter was activated under ABA treatment, with strong GFP expression in tobacco leaves expressing the transformed *CdDHN4* promoter (PBCdDHN4-P::GFP). Meanwhile, a drought-response element (DRE), which is the main feature of the ABA-independent regulatory pathway, was not detected in the *CdDHN4* promoter sequence. These results imply that the *CdDHN4* is regulated by an ABA-dependent signal pathway under drought condition.

Y_n_SK_m_-type dehydrins are strongly induced by drought and salt and are associated with plant stress tolerance (Graether and Boddington, 2014). They are expressed in the leaves and roots of ABA-treated seedlings. Here, *CdDHN4* belongs to the YSK_2_ subfamily (data not shown) and was up-regulated by drought, salt stress, low temperatures and high-temperatures (Figure 3). However, the level of *CdDHN4* transcription were greatly increased under drought and cold conditions compared to other two stress conditions at 10 and 15 d. Moreover, the dehydrin promoter::GFP reporter was associated with a strong GFP signal during transient expression in transgenic tobacco leaves exposed to drought and ABA addition, while no GFP signal was detected under normal conditions (control) (Figure 6). Many studies have shown that drought stress can induce ABA expression in plants. Thus, drought might indirectly activate the promoter of *CdDHN4* through the ABA signal pathway. The activation of the *CdDHN4* promoter was in accordance with the expression of *CdDHN4* under exposure to 5 or 50 μM exogenous ABA. The variation in *CdDHN4* expression was positively correlated with the changes in levels of endogenous ABA following ABA addition (Figures 4B,C). Taken together, these results indicate that *CdDHN4* is up-regulated by drought stress via an ABA-dependent pathway.

The low temperature-responsive element (LTRE), with the core sequence CCGAC, is critical to the low-temperature response. Previous studies have demonstrated that direct mutation of the CCGAC core sequences in the TGGCCGAC repeat of a native promoter resulted in loss of low-temperature response and the LTRE, which is closer to the TATA box, had a more pronounced effect on regulating the low-temperature expression of the *BNI15* gene (Jiang et al., 1996). In this study, one TATA box and two G-box (core sequences CACGTG) were near the LTRE region of the *CdDHN4* promoter (Figure 5) and the *CdDHN4* promoter was weakly activated by low temperatures compared to ABA treatment (Figure 6). However, *CdDHN4* expression was greatly up-regulated by low temperature, indicating that cold-induced high *CdDHN4* expression is associated with the *cis*-regulatory element LTRE in the *CdDHN4* promoter, but its regulation may involve another signal pathway that is independent of ABA.

Electrolyte leakage (EL) and relative water content (RWC) in leaves are often used as indicators of plant injury under abiotic stresses. The RWC and Fv/Fm in Tifway leaves were significantly lower under drought stress than under the other four treatments at 12 d, while EL was much higher than under the other four treatments, indicating that Tifway was more sensitive to drought than to heat, cold and salt stresses. In addition, the RWC was much higher in Tifway than the C299 under drought conditions at 5, 10, and 15 d, while the *EL*-value was significantly lower in Tifway than in C299, which was further demonstrating that C299 was more sensitive than Tifway to drought stress.

The expression level of *CdDHN4* is closely associated with ability of the plant to tolerate. In this study, *CdDHN4* expression levels increased with drought stress in leaves, stems and roots and were much higher in leaves than in stems and roots (Figure 2). Interestingly, roots are the first plant tissue that encounter drought conditions, but the level of *CdDHN4* transcription in roots was lower than in the other two tissues, possibly because of the rapid drought response to ABA in the leaves, closing the stomata and inducing the expression of dehydrin genes (BRAY, 1988). In addition, higher expression of *CdDHN4* in leaves was found in Tifway than in C299 under drought condition, a finding in agreement with previous studies in which dehydrin gene expression levels were higher in drought-tolerant genotypes than in drought-sensitive ones (Lopez et al., 2003; Hu et al., 2010; Zhou et al., 2014) and dehydrin-protein expression was positively associated with plant drought tolerance (Hu et al., 2010). The expression of the dehydrin gene in leaves is positively related to drought tolerance.

ABA strongly induced dehydrin gene expression under drought conditions, and there is a close relationship between ABA accumulation and the level of dehydrin expression in the response to drought stress (Deng et al., 2005; Allagulova et al., 2007; Qin and Qin, 2016). *CdDHN4* was up-regulated by 5 or 50 μM exogenous ABA in both bermudagrass genotypes, however, the sensitivity of *CdDHN4* to exogenous ABA was higher in Tifway than in C299. In addition, ABA accumulation in leaves increased quickly and was higher in Tifway than in C299 after 6 h of ABA treatment and then greatly decreased to a level lower than that in C299. The high sensitivity of *CdDHN4* to ABA may help Tifway adapt to drought conditions, thus, preventing ABA over-accumulation.

ABA modulates many aspects of plant growth and development including seed germination, embryo maturation, leaf senescence, stomatal aperture, and adaptation to environmental stresses (Shinozaki and Yamaguchi-Shinozaki, 2006; Wasilewska et al., 2008). Low-level ABA accumulation is necessary for regulating plant development and healthy growth; however, high-level ABA accumulation is a senescence accelerator (Gepstein and Thimann, 1980), that promotes leaf abscission and senescence (Breeze et al., 2011; Liang et al., 2014). Huang et al. reported that heat-induced leaf senescence in bentgrass was associated with changes in three major senescence-related hormones: ethylene, abscisic acid, and cytokinins (Huang and Xu, 2007). Exogenous application of ABA accelerates the senescence of detached leaves, in which high levels of ABA is a stress condition. In this study, the lower sensitivity of *CdDHN4* to ABA in C299 than in Tifway caused higher ABA accumulation at 12 h and 24 h of exogenous application of ABA. On the other hand, the level of ABA was must higher in C299 than in Tifway after 10 d of drought stress. Excess ABA accumulation in C299 may accelerate leaf senescence, which was demonstrated by higher EL and lower RWC in C299 than in Tifway under drought stress (Table 2). Thus, the sensitivity of *CdDHN4* to ABA might be an important determinant of the drought tolerance of bermudagrass.

The dehydrin genes in plants respond significantly to abiotic stress and are associated with high drought tolerance. The *CdDHN4* promoter contained the main *cis*-regulatory elements involved in ABA-dependent gene expression, including one ABRE, one MBS and one MYC near the TATA-box. As a result, the *CdDHN4* promoter was activated by ABA addition and drought, and *CdDHN4* was up-regulated in the two bermudagrasses via an ABA-dependent pathway under drought condition. The expression of *CdDHN4* and sensitivity of *CdDHN4* to ABA were higher in Tifway than in C299, which might result in a lower EL level and a higher RWC level in the leaves of Tifway than in C299. The sensitivity of *CdDHN4* to ABA may play an important role in avoiding ABA over-accumulation in Tifway under drought conditions.

In addition, the 5′ region upstream of *CdDHN4* in C299 was extremely similar to that of Tifway (Supplement Figure 2), however, the expression levels of DHN differ under the same exogenous ABA treatment and under the same drought-stress conditions. We hypothesize that this difference could be attributable to differences in the efficient of transcription factors (TFs), in the number of genomic copies of the dehydrin gene or in methylation sites in the promoter. These possibilities should be studied further.

## Author contributions

AL, YA, and PZ designed research; AL and NF performed research; AL, JX, and SY analyzed data and discussed results; AL wrote the paper; YA and PZ revised the paper. All authors contributed to improving the paper and approved the final manuscript.

### Conflict of interest statement

The authors declare that the research was conducted in the absence of any commercial or financial relationships that could be construed as a potential conflict of interest.
