# Supplementary material for: Expression of CdDHN4, a Novel YSK2-Type Dehydrin Gene from Bermudagrass, Responses to Drought Stress through the ABA-Dependent Signal Pathway
Source: Front Plant Sci. 2017 May 16;8:748. doi: 10.3389/fpls.2017.00748 (PMC5433092; doi:10.3389/fpls.2017.00748)
Supplement: Supplementary file 1 [file Image1.pdf]

Supplementary Material

Expression of *CdDHN4*, a novel YSK_2-type Dehydrin gene from Bermudagrass, responses to drought stress through ABA-dependent signal pathway

First author: Aimin Lv

***Correspondence:**
Corresponding Author: Peng Zhou: e-mail pzhou0063@sjtu.edu.cn; Yuan An: e-mail anyuan@sjtu.edu.cn

Supplementary Figures

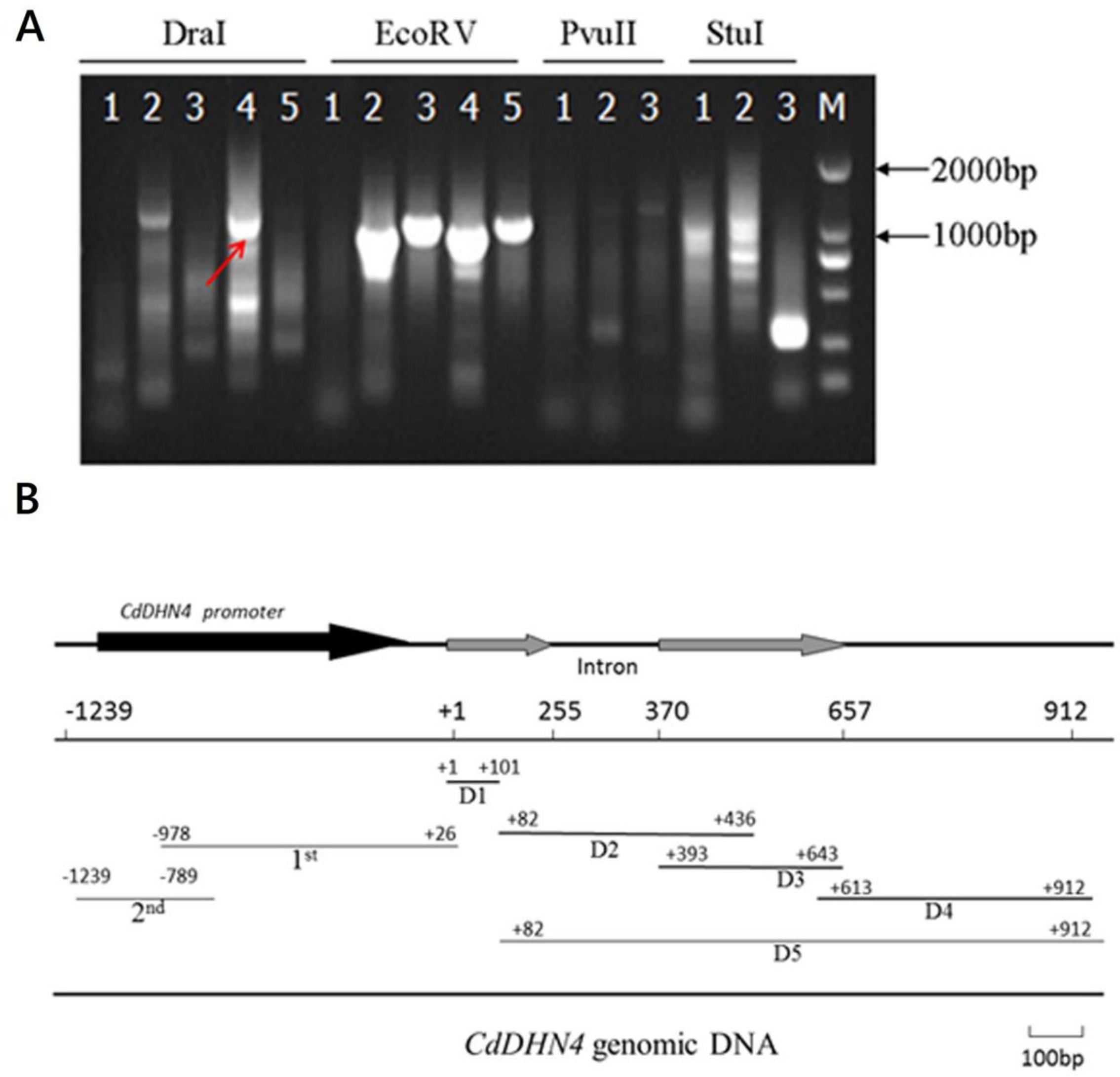

Supplement Figure 1. Genomic structure and upstream promoter of *CdDHN4*.

A-The electrophoresis represent results of upstream of *CdDHN4* with thermal asymmetric nested PCR. The Genomic DNA was digested with *Dra*I, *EcoR*V, *Pvu*II and *Stu*I. Based on the four libraries, gene-specific primers(PR1, PR2 and PR3 (Table 1) and AP1 primer (outer Adapter Primer, provided by the kit) were used to amplify the 3'- and 5'-flanking region, respectively. M-DL2000，1-1st PCR，2-2nd PCR，3-2nd PCR, primer with only AP1, 4-3rd PCR，5-3rd PCR, primer with only AP1.

B-Genomic structure and cloning of *CdDHN4*. Genomic sequence was assembled by DNA fragments that can overlap the whole *CdDHN4* sequence. Those DNA fragments were amplified by PCR based on *CdDHN4* cDNA sequence.
