# Supplementary material for: Expression of CdDHN4, a Novel YSK2-Type Dehydrin Gene from Bermudagrass, Responses to Drought Stress through the ABA-Dependent Signal Pathway
Source: Front Plant Sci. 2017 May 16;8:748. doi: 10.3389/fpls.2017.00748 (PMC5433092; doi:10.3389/fpls.2017.00748)
Supplement: Supplementary file 2 [file Image2.pdf]

```
C299-promoter      GTAGTTTCGTCTGAGTGTTGCGTAATTATTTTACGGCATAAAAAGTACATAAACTACTAAAATTTTCACAGTAATTATTA      80
Tifway-promoter    GTAGTTTCGTCTGAGTGTTGCGTAATTATTTTACGGCATAAAAAGTACATAAACTACTAAAATTTTCACAGTAATTATTA      80
Consensus          gtagtttcgtctgagtgttgcgtaattattttacggcataaaaagtacataaactactaaaattttcacagtaattatta

C299-promoter      TCAGTAGCATATATTCTTTCGGAAAAAAATAACCATCGAAAAAA.TACTGAAGTTTTATTCAGTAATTTCAGTTTTGGTGT     159
Tifway-promoter    TCAGTAGCATATATTCTTTCGGAAAAAAATAACCATCGAAAAAAATACTGAAGTTTTATTCAGTAATTTCAGTTTTGGTGT     160
Consensus          tcagtagcatatattctttcggaaaaaaataaccatcgaaaaaa tactgaagttttattcagtaatttcagttttggtgt

C299-promoter      AGAATAACTATTCTACACCCTAGATGTAGAATAGCATATGGCTATATATATATATATATATAT....ACTGTGATTTGAA     235
Tifway-promoter    AGAATAACTATTCTACACCCTAGATGTAGAATAGCATATGGCTATATATATATATATATATATATATACTGTGATTTGAA     240
Consensus          agaataactattctacaccctagatgtagaatagcatatggctatatatatatatatatatat    actgtgatttgaa

C299-promoter      TGTGATTTGAATTTGAAATATGTGGTTTTTAAATTATTATAAATATAAATACATTTAATATCCCAGTGACGGATAACATG     315
Tifway-promoter    TGTGATTTGAATTTGAAATATGTGATTTTTAAATTATTATAAATATAAATACATTTAATATCCCAGTGACGGATAACATG     320
Consensus          tgtgatttgaatttgaaatatgtg tttttaaattattataaatataaatacatttaatatcccagtgacggataacatg

C299-promoter      ATGTTACCACGTCATCGCTGGGAAACCATCGCAGTGGCGGCGTCGTGATGTCACCTCATTCGCCTCTAAGATTATTTTAC     395
Tifway-promoter    ATGTTACCACGTCATCGCTGGGAAACCATCGCAGTGGCGGCGTCGTGATGTCACCTCATTCGCCTCTAAGATTATTTTAC     400
Consensus          atgttaccacgtcatcgctgggaaaccatcgcagtggcggcgtcgtgatgtcacctcattcgcctctaagattattttac

C299-promoter      CAGCGGCGGTTTCTGTGACGTCGCCACGCGGTCACTGGGGTTAACGTTCCAGTGGCAACGTGGCTTATGTCACCGCTTCG     475
Tifway-promoter    CAGCGGCGGTTTCTGTGACGTCGCCACGCGGTCACTGGGGTTAACGTTCCAGTGGCAACGTGGCTTATGTCACCGCTTCG     480
Consensus          cagcggcggtttctgtgacgtcgccacgcggtcactggggttaacgttccagtggcaacgtggcttatgtcaccgcttcg

C299-promoter      CCACTGGGGTAAATATCCCAGTGGTGATTGGTGTGATATCACCAGTGCGCTACCAGGAAAATATTCCAGCGGCGGATCTG     555
Tifway-promoter    CCACTGGGGTAAATATCCCAGTGGTGATTGGTGTGATATCACCAGTGCGCTACCAGGAAAATATTCCAGCGGCGGATCTG     560
Consensus          ccactggggtaaatatcccagtggtgattggtgtgatatcaccagtgcgctaccaggaaaatattccagcggcggatctg

C299-promoter      GTGTCATCGCCCAACTGGCACCCAGTTCAGATATGCTCAGATATATGCAGATCAACAAATTCATCTAGGTCTATATTGGT     635
Tifway-promoter    GTGTCATCGCCCAACTGGCACCCAGTTCAGATATGCTCAGATATATGCAGATCAACAAATTCATCTAGGTCTATATTGAT     640
Consensus          gtgtcatcgcccaactggcacccagttcagatatgctcagatatatgcagatcaacaaattcatctaggtctatattg t

C299-promoter      TGATGTTTCTTTCTTTTTTGTCATAATTTTTTACATTGATCCTTATTTTATCACTAACTTATTCTTCAGAGGGTTCTCTA     715
Tifway-promoter    TGATGTTTCTTTCTTTTTTGTCATAATTTTTTACATTGATCCTTATTTTATCACTAACTTATTCTTCAGAGGGTTCTCTA     720
Consensus          tgatgtttctttcttttttgtcataattttttacattgatccttattttatcactaacttattcttcagagggttctcta

C299-promoter      TCAAAATATCTACTGTGTACTGCTCTTTTCATTCTGAATTACACCTTATACCACTTGGTAACATGGTTGCACACATGCAT     795
Tifway-promoter    TCAAAATATCTACTGTGTACTGCTCTTTTCATTCTGAATTACACCTTATACCACTTGGTAACATGGTTGCACACATGCAT     800
Consensus          tcaaaatatctactgtgtactgctcttttcattctgaattacaccttataccacttggtaacatggttgcacacatgcat

C299-promoter      GGTTGGTAATTTCGTAAAGCAGAGATAGTACTTGGTATTTATCCGAACCTAGTTCAGATATTTTTCCAGTACGTAGAAGC     875
Tifway-promoter    GGTTGGTAATTTCGTAAAGCAGAGATAGTACTTGGTATTTATCCGAACCTAGTTCAGATATTTTTCCAGTACGTAGAAGC     880
Consensus          ggttggtaatttcgtaaagcagagatagtacttggtatttatccgaacctagttcagatatttttccagtacgtagaagc

C299-promoter      CAGAGTTGGATAAGACCAAAAACTCAAACCTGCAACTTCCAGAAACCGCCGCCACCCACGTAAACCCCTCCTGGTCTCCA     955
Tifway-promoter    CAGAGTTGGATAAGACCAAAAACTCAAACCTGCAACTTCCAGAAACCGCCGCCACCCACGTAAACCCCTCCTGGTCTCCA     960
Consensus          cagagttggataagaccaaaaactcaaacctgcaacttccagaaaccgccgccacccacgtaaacccctcctggtctcca

C299-promoter      TTTTCCCCCGCGTGTAGGCGCATGCAGAATCGTTATCCTGTTCTTGTCTCCTCTCAGCTCGCCGCGTCACGGTCCGACAG    1035
Tifway-promoter    TTTTCCCCCGCGTGTAGGCGCATGCAGAATCGTTATCCTGTTCTTGTCTCCTCTCAGCTCGCCGCGTCACGGTCCGACAG    1040
Consensus          ttttcccccgcgtgtaggcgcatgcagaatcgttatcctgttcttgtctcctctcagctcgccgcgtcacggtccgacag

C299-promoter      TTTCCAGAAGCCCTCGCGACGTGGCGCCGCTGACACGCCTGCACACGTCGCGCAACACTTCGCCGTCGAACATCTATAAA    1115
Tifway-promoter    TTTCCAGAAGCCCTCGCGACGTGGCGCCGCTGACACGCCTGCACACGTCGCGCAACACTTCGCCGTCGAACATCTATAAA    1120
Consensus          tttccagaagccctcgcgacgtggcgccgctgacacgcctgcacacgtcgcgcaacacttcgccgtcgaacatctataaa

C299-promoter      TGGCGTCACTTCCTCATCACCAAAGTTCCACAGAAATCCAGTAACAAGCACACTAAAACAAGTCGACAACACTTGCGGTT    1195
Tifway-promoter    TGGCGTCACTTCCTCATCACCAAAGTTCCACAGAAATCCAGTAACAAGCACACTAAAACAAGTCGACAACACTTGCGATT    1200
Consensus          tggcgtcacttcctcatcaccaaagttccacagaaatccagtaacaagcacactaaaacaagtcgacaacacttgcg tt

C299-promoter      TGGGTGTGTGAGAGAGCAGCTTAGTAGTCAGTAGTCACC                                             1234
Tifway-promoter    TGGGTGTGTGAGAGAGCAGCTTAGTAGTCAGTAGTCACC                                             1239
Consensus          tgggtgtgtgagagagcagcttagtagtcagtagtcacc
```

Supplement Figure 2. The upstream sequence of *CdDHN4* between ‘Tifway’ and ‘C299’

Promoter sequence of *CdDHN4* in ‘C299’ was obtained according to the known sequence of ‘Tifway’. DNAMAN(Version 5.2) sequence alignment showed the identity was 99.36%.
